# Mental well-being model of Thai older adults: Comparing the aging in place and aging migrant groups

**DOI:** 10.1371/journal.pone.0311284

**Published:** 2024-12-12

**Authors:** Arunya Tuicomepee, Juthatip Wiwattanapantuwong, Panrapee Suttiwan, Rewadee Watakakosol, Sompoch Iamsupasit, Phummaret Phupha

**Affiliations:** 1 Faculty of Psychology, Chulalongkorn University, Bangkok, Thailand; 2 Center of Excellence in Universal Design, Chulalongkorn University, Bangkok, Thailand; 3 Faculty of Education, Burapha University, Saen Suk, Chon Buri, Thailand; Mexican Social Security Institute: Instituto Mexicano del Seguro Social, MEXICO

## Abstract

**Objective:**

Although aging well and aging in place policies have been encouraged in many countries, a consistent challenge is insufficient evidence on older migrants. This study compared mental well-being models of older adults between aging in place and aging migrant groups. The model included social factors (e.g., social well-being) and personal factors (e.g., cognitive function and psychological distress).

**Methods:**

Participants were 334 Thais (187 aging in place, 147 aging migrants). Their mean age was 69.79 ± 7.28 years old. All met the selection criteria: age 60 years or over, voluntary participation, and ability to visit the community area for study participation. Instruments were *The Modified Mini-Mental State (3MS)*, *21-item Depression*, *Anxiety*, *Stress Scale*, *15-item Social Well-Being Scale*, *and 5-item WHO Well-being Index*. Data were collected in the five regions of Thailand from March 2021 to February 2022. Local psychologists and research assistants assessed and interviewed the participants. Maximum likelihood with the Satorra-Bentler correction was used for parameter estimation.

**Results:**

Both models fit the empirical data. The multi-group model estimation yielded a difference between the models. The factors of the aging in place group that were associated with mental well-being were psychological distress (SE = 0.08, p < .001) and cognitive function (SE = 0.07, p < .001), but not social well-being (SE = 0.09, ns). For the migrant group, psychological distress (SE = 0.12, p < .01) and social well-being (SE = 0.11, p < .001) were significant predictors of mental well-being, but not of cognitive function (SE = 0.12, ns).

**Conclusions:**

By understanding differences in the mental well-being models between the two groups, policy makers and service providers can better address the needs of older adults. Policies and programs should be designed to reduce their psychological distress. Maintaining social connections can be crucial for migrants, while activities such as lifelong learning and physical activities to enhance cognitive function can benefit people who are aging in place.

## Introduction

Ongoing gerontology research on positive aging and aging in place continues to contribute valuable knowledge on how to support older adults in maintaining mental well-being, social relationships, independence, and quality of life [1–3]. The World Health Organization (WHO) defines “aging in place” as “[older people’s] ability to remain at home in their familiar surroundings and maintain the relationships that are important to them” [4]. Rowles demonstrated how older people who have resided in the same community for a long time develop a sense of attachment through a lifelong accumulation of experiences in that place, which can give them a sense of identity [5]. Empirical studies on Rowles’s concept of place attachment emphasize the importance of maintaining emotional and social connections to one’s living environment as a critical aspect of aging in place. Research on aging in place highlights that place attachment contributes significantly to mental well-being by providing emotional comfort, a sense of belonging, and continuity [1–6]. Among older adults with a migration history, either within or between countries, the experience of aging and mental well-being can be different. Aging migrants may face challenges and opportunities related to relocation such as social isolation, loss of access to familiar community groups, religious institutions, or social organizations, and difficulties adjusting to new environments [6].

Thailand is among the most rapidly aging countries in the world. Its population aged 60 years and over will comprise 28% of the total population by the 2030s [7]. In addition, Thailand’s the elderly population is growing not only in numbers but also in diversity. Internal or domestic migration is ’’the movement of people from one part of a country to another” [8]. Approximately9.4% of the Thai population have migrated within the country over 5 years [9]. Thai older adults who are internal migrants tend to move from rural to urban areas or from one region to reunite with family members, for family resettlement, or to find a better place to live [10]. As Thailand’s elderly population increases, there has been more support for aging in place instead of institutional living [11].

However, previous studies on the mental well-being for older Thai adults and those with a migration history have been inconclusive. Pekalee and Gray examined happiness as an indicator of mental well-being among 7, 829 Thais 75 years of age and older. They found that home environment both had a direct impact on their happiness, but also moderated the relationship between physical disability and happiness. Their findings support the benefit of allowing older adults to age in place [10]. Thongsrikate examined happiness in older adults who relocated within Thailand. Findings revealed that these migrants demonstrated a high level of happiness and mental well-being, especially if they had a variety of familial and social resources compared to those without those resources [12].

Mental well-being is guided by the premise of positive aging that emphasizes mental, emotional, and physical well-being. It suggests that aging should be viewed and experienced positively [13]. According to the WHO, Mental well-being can be defined as an individual’s ability to develop their potential, work productively and creatively, build strong and positive relationships with others, and contribute to their community [14]. Mental well-being and its associated factors among older adults are of increased interest to scholars [14–16]. In Thailand, Tuicomepee and colleagues examined mental well-being and its predictors among 107 older residents of the Bangkok metropolitan area. The predictors included personal factors such as psychological distress (e.g., depression, anxiety, stress), future time perspective, and social well-being. Data collection was completed before the COVID-19 pandemic. Multiple regression was used to analyse the data. Findings revealed that all predictors significantly predicted Thai older adults’ mental well-being and accounted for 32.7% of total variance. The most salient predictors were depression, social well-being, and future time perspective. However, stress and anxiety were not significant predictors of mental well-being [16]. Cresswell‑Smith and colleagues reviewed empirical studies on the determinants of mental well-being in older adults. The studies were published between 2003 and 2018 and found in databases such as Web of Science, EBSCOhost, and Google Scholar. Cresswell‑Smith and colleagues noted that social factors such as satisfaction with their social network and meaningful social activities were key determinants of older adults’ mental well-being [17]. Supporting Keyes and Shapiro, they claimed that older adults’ social well-being, their assessments of their experiences in society, benefits their health and mental well-being [18]. Keyes’ concept of social well-being addresses the evaluation of individuals in their situation and in society with five dimensions: 1) social integration; 2) social acceptance; 3) social contribution; 4) social actualization; and 5) social coherence [19]. It is meaningful to examine the extent to which social well-being predict the mental well-being of aging in-place among older Thais.

Despite considerable research on social and environmental factors, empirical evidence has revealed that cognitive function and psychological distress (e.g, depression, anxiety, stress) are associated with older people’s mental well-being. Cognitive function refers to mental processes involved in acquiring knowledge, manipulating information, and reasoning [20]. Previous studies on cognitive function among older adults provide evidence linking greater cognitive function with well-being [15, 21]. Finlay and colleagues highlighted the potential role of neighbourhood social infrastructure in enhancing cognitive function and protecting against cognitive decline among adults who are aging in place. Neighbourhood social infrastructure included restaurant, senior centre, and civic and social groups where older adults regularly meet. Finlay and colleagues found a moderate impact of access to neighbourhood sites on cognitive function in older adults. Hower, they did not find that access to food and drinking place was significantly associated with cognitive function [21]. Many older adult migrants contend with decreasing social network and support. Shankar and colleagues found social isolation among migrants to be a risk factor for cognitive impairment and cognitive decline [22]. Some studies showed that older migrants demonstrated poorer cognitive function than non-migrants in hosting places [23], while other studies indicated no association between migration and cognitive function [24]. Psychological distress among older adults has also been the topic of several studies as an indicator of well-being in older adults. Psychological distress consists of non-specific symptoms of stress, anxiety, and depression and is a widely used indicator of mental health and well-being in surveys of population health and aging [25]. The prevalence psychological distress tends to be higher among migrants [26]. It is possible that people who are aging in place would benefit from personal, social, and environmental resources to promote mental well-being. However, aging migrant populations may not benefit in the same way.

This study provides a predictive mental well-being model for older adults based on the premise that positive aging and place attachment are linked. By integrating personal and social factors, the mental well-being model includes personal factors such as cognitive function, psychological distressand social factors such as social well-being. The purpose of this study is to examine differences between the mental well-being models of older adults who are aging in place and those who are internal migrants. An evidence-based study of mental well-being models including the association between these two groups may inform policy makers and practitioners on how to implement aging in place policies that allow older adults to thrive while maintaining their mental well-being and quality of life.

## Method

This research is a part of Subproject No.3 under the interdisciplinary aging research plan “Appropriate Housing for Thai Elderly to Promote Physical and Mental Health by Age- Friendly Community Concept”, which examined the health and well-being of community-dwelling older adults. Participants were selected by Quota sampling as follows. Five provinces representing Thailand’s five regions were selected: Bangkok, the Northern region (Chiang Mai Province), the Northeastern region (Khon Kaen Province), the Central region (Pathum Thani Province), and the Southern region (Songkhla Province). Two communities were nominated by provincial governors based on two criteria 1) more than 1,000 healthy adults aged 60 or older; and 2) community leaders or a team that are in contact with those older adults.

After the community was chosen, the researcher contacted the community leader, discussed the date of visit and the local COVID-19 situation. The project was then announced through community hotline to recruit participants who meet the selection criteria. A list of proposed participants was compiled before data collection began.

### Participants

To represent the population of community-dwelling older adults, the approximate sample size per community was 20–50 or 2–10% of healthy older adult population. This study did not cover residents of institutions or nursing homes. The older people with severe mobility concerns or daily activity limitations are not the target of this study. Eligible participants fulfilled the following inclusion criteria: (a) aged 60 years or over; (b) participating voluntarily; and (c) able to visit the community area to participate in the study. Therefore, bedridden or cognitively impaired participants, those who were absent on the date of data collection or who did not consent to participate were excluded. For participants who met the criteria but had difficulties in transportation, the researcher offered free roundtrip bus tickets.

In this study, an "aging in place" older adult refers to older people who remain in the same community where they were born and raised, maintaining long-term connections, resources, and support networks within that community throughout their lives. This concept reflects the deep place attachment described by Rowles. Conversely, an "aging migrants" older adult refers to older adults who have relocated from another part of Thailand to live in a new community later in life, after establishing their primary relationships and resources elsewhere, regardless of their current length of residence. To determine a participant’s status, we asked, “Were you born and raised in this community?” Those who answered “yes” were classified as aging in place, while those who answered “no” were classified as aging migrants. Aging migrants were then asked a follow-up question: “How many years have you been living in this community?” In our sample, there were 187 aging in-place older adults and 147 aging migrant older adults. The length of residence for aging migrants ranged from 2 to 70 years. The 97-year-old participant with a 70-year migration duration moved to the community around age 27, having spent a significant portion of his life elsewhere before relocating. Therefore, his situation aligns more closely with the aging migrant classification rather than the traditional aging in place definition.

In total, 334 older adults across five regions participated in the study (Table 1). Their mean age was 69.79±7.28 years old (ranging from 60 to 97). There were more females than males (73.5% vs. 26.5%). The mean duration of migration was 32.44 ±17.90 years. Most migrated from rural to urban areas for settlement and job opportunities. The older adults varied in education, marital status, and level of functionality. Table 1 gives their demographic details.

**Table 1:** Demographic data of older adult aging in place and migrant groups.

	Aging in Place Group (n = 187)	Aging Migrant Group (n = 147)	All (N = 334)
Age			
60–69	108(54.8)	79(53.7)	187(54.4)
70–79	62(31.5)	54(36.7)	116(33.7)
80 and above	27(13.7)	14(9.5)	41(11.9)
Gender			
Female	130(66.0)	112(76.2)	242 (70.3)
Male	67(34.0)	35(23.8)	102 (29.7)
Areas			
Central (Pathum Thani province)	27(13.7)	40(27.2)	67(19.5)
Northeastern region (Khon Kaen Province	67 (34.0)	15(10.2)	82(23.8)
Northern region (Chiang Mai province),	74(37.6)	2(1.4)	76 (22.1)
Southern (Songkhla province)	18(9.2)	60(40.8)	78(22.6)
Bangkok	30(20.4)	11(5.6)	41 (11.9)
Duration of Living in the current community of the migrants (year) Mean = 32.44, SD = 17.90, Mdn = 32, Min = 2, Max = 70			

### Measures

The following measures were used to collect data.

1. The Modified Mini-Mental State Examination (3MS). Teng and Chui [27] developed the 3MS to assess a person’s cognitive abilities (e.g., attention, concentration, orientation to time and place, long-and short-term memory, language ability, constructional praxis, abstract thinking, and list-generating fluency). The Thai version was developed by the researcher. The 3MS is a 15-item test that takes 5 to 10 minutes to complete. The 3MS consists of three components: 1) learning, recall and executive function; 2) psychomotor ability and expressive language; and 3) orientation and awareness [28]. In this study, local psychologists and research assistants were trained to administrate the 3MS. The training composed of the manual of how to ask question and scoring. A 60-minute online training session was set up to confirm their understanding in the test. The total score ranged from 0 to 100. Teng and Chui reported that the original 3MS had a Cronbach’s alpha coefficient of 0.87. For this study, the 3MS yielded a Cronbach’s alpha coefficient of 0.84.
2. The Thai version of the Depression, Anxiety and Stress Scale—21 Items (DASS-21). The Thai version of the DASS-21 [29] measured depression, anxiety and stress among older adults during the prior two weeks. Each of the three DASS-21 scales contains seven items, divided into subscales. The scales use a 4-point rating scale (0 = “did not apply to me at all” to 3 = “applied to me very much or most of the time”). The total scores ranged from 0 to 63. For this study, the Thai version of the DASS-21 yielded a Cronbach’s alpha coefficient of 0.93.
3. The Social Well-Being Scale (SWBS). The SWBS, 15-item measure of social acceptance, social coherence, social actualization, social integration, and social contribution was developed by Keyes [19]. The researchers developed the Thai version. Each item is rated on a 5-point scale (1 = strongly disagree to 5 = strongly agree). A total score is calculated by adding all items. The Thai version yielded a Cronbach’s alpha coefficient of 0.71.
4. The Thai version of the WHO-5 Well-being Index (WHO-5). The Thai version of the WHO-5 [30] was employed to measure mental well-being. The Thai version was created by Tuicomepee and colleagues [16]. It had two constructs: 1) positive functioning (three items); and 2) psychological well-being (two items). Participants were asked to rate themselves during the past two weeks from 0 = never to 5 = all the time. Scores ranged from 0 (absence of well-being) to 5 (maximal well-being). The Thai version of the WHO-5 yielded a Cronbach’s alpha coefficient of 0.81.

Translation. All measures, except the WHO-5 and DASS-21, were translated from English to Thai. The WHO-5 and DASS-21 were translated into Thai with established psychometric properties. The researcher translated the English version of the measures into Thai. The Thai version was then translated back into English by bilingual clinical psychologists. The original and back-translated English versions of the questionnaires were rated by two other bilingual experts in English and Thai. The experts were asked to rate their agreement with the translated version to ensure equality and comparability in meaning and ease of understanding. Inter-rater reliability of the questionnaires was 100%, with both experts agreeing that the Thai version was sufficiently similar to the English version. The item contents and meanings remained mostly unchanged throughout the translation process.

### Procedure

Before data collection, ethical approval was obtained from the Ethics Review Committee for Research Involving Human Subjects, Health Science Group, Chulalongkorn University (No.231.1/2563). Data were collected in the five regions between March 2021 and February 2022. The research team developed guidelines and video clips on data collection and specific assessment of older adults, in addition to training the research team, psychologists, and research assistants in all five regions to meet the required standards for data collection.

An age-friendly version of consent form was prepared and volunteers at the registration desk explained the research objective, procedure, possible benefit and risk for to prospective participants before they agreed to participate and signed the consent form before data collection started. Participants who could not read and write were supported by the volunteer, and observed by local community staffs. They gave a fingerprint instead of signature.

Our study on active aging focuses on individuals who are actively engaged in daily activities. To ensure the relevance of our research, we excluded those who were unable to participate actively. This criterion helps align our participants with the study’s emphasis on active aging. We also excluded older adults with severe mobility issues or significant limitations in daily activities, as these conditions could hinder adherence to study protocols and task completion, potentially leading to data inconsistencies and complicating result interpretation. During the recruitment process, participants with severe mobility issues, hearing impairments, or communication difficulties were excluded at the registration desks. Data collection took about one hour per participant. Upon completion, participants received a small gift bag, which included a project T-shirt and a lunch box.

The COVID-19 pandemic in Thailand was declared in January 2020. During data collection from March 2021 to February 2022 the Thailand Ministry of Public Health proposed the bubble and seal measure to reduce the burden on the public health system and to enable the business and government sectors to continue normal operations. The bubble and seal measure made it possible for the research teams, local stakeholders, and older adults to participate in the study. The research teams complied with all disease prevention and control measures.

The data collection protocol was modified as follows: 1) data were collected only by local psychologists and researchers to reduce the risk of COVID-19 exposure; 2) cooperation with local staff was closely monitored; 3) the number of older adults undergoing mental health screenings was reduced from 40 to 20 per day (i.e., 10 in the morning and 10 in the afternoon); and 4) the country protocol for COVID-19 prevention [31] was followed. Everyone at the site had their temperature taken, wore masks, and sanitized their hands before and during data collection.

### Data analysis

**Fig 1:**
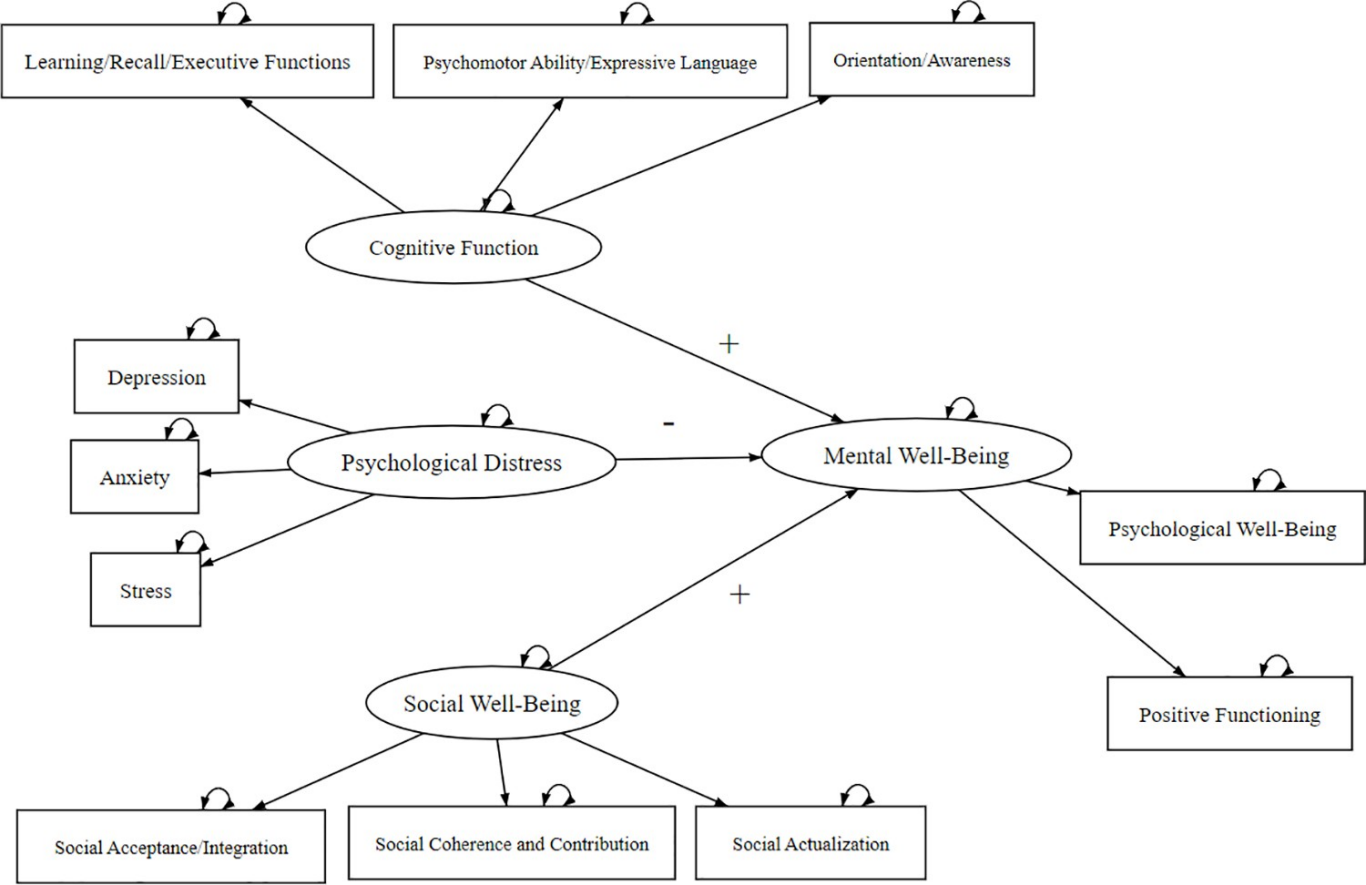
The conceptual model of this study.

The conceptual model depicted in Fig 1 examines the differences in mental well-being between older adults aging in place and those in aging migrant groups. In this model, mental well-being is the dependent variable, while social factors (such as social well-being) and personal factors (such as cognitive function and psychological distress) serve as independent variables.

For parameter estimation, maximum likelihood with the Satorra-Bentler correction (denoted by MLSB [32, 33] was employed. MLSB is known to accommodate nonnormality of the data and generate scaled chi-square index and robust standard error estimates [34, 35]. To evaluate the goodness-of-fit, the following fit indices were considered: scaled chi-square (χ2), comparative fit index (CFI), Tucker-Lewis index (TLI), (SRMR), and the root mean square error of approximation (RMSEA) [35]. The cutoff values for evaluating model fit include the CFI and TLI were greater than or equal to 0.95, and the SRMR and RMSEA were less than 0.08 [32, 33].

## Result

The mean and standard deviation of each variable and zero-order correlations between the four latent variables were calculated. Table 2 indicates each variable’s descriptive statistics for the aging in place and aging migrant groups. In terms of cognitive function, the aging in place group demonstrated lower scores on Learning/recall/executive function and Psychomotor ability/ expressive language dimensions compared to the aging migrant group, but not the Orientation/awareness dimension. For psychological distress, both groups had comparable levels of depression, anxiety, and stress. However, in termed of social and mental well-being, the aging in place group exhibited higher scores than the aging migrant group. Table 3 shows correlations between the variables.

**Table 2:** Descriptive statistics for the study variables.

	Min-Max	Aging in Place Group (n = 187)	Aging Migrant Group (n = 147)	All (N = 334)
		Mean (SD)	Mean (SD)	Mean (SD)
1. Cognitive function				
Learning/recall/executive function	4–55	34.70 (10.24)	34.77(11.33)	34.66(10.78)
Psychomotor ability/ expressive language	0–49	14.59 (9.29)	14.95 (8.74)	14.75(8.03)
Orientation/awareness	2–47	17.02 (9.34)	14.56 (3.89)	15.93 (7.54)
2. Psychological Distress				
Depression	0–3	0.54 (0.60)	0.54 (0.66)	0.54 (0.62)
Anxiety	0–3	0.56 (0.56)	0.54 (0.59)	0.55(0.57)
Stress	0–3	0.66 (0.63)	0.66 (0.63)	0.66(0.64)
3. Social Well-being				
Social acceptance and integration	4–10	7.88 (1.29)	7.54 (1.17)	7.73(1.25)
Social coherence and contribution	3–10	7.40 (1.23)	7.20 (1.10)	7.31(1.17)
Social actualization	1–5	3.58 (0.90)	3.20 (0.83)	3.41(0.89)
4. Mental Well-Being				
Psychological well-being	0–5	3.35 (1.27)	3.34 (1.23)	3.35(1.25)
Positive functioning	0–5	3.70 (1.30)	3.61 (1.23)	3.66(1.27)

**Table 3:** Correlations among the variables for aging in place group and (n = 187) and aging migrant group (n = 147).

1.Cognitive function (alpha = .67(.52))	1.1	1.2	1.3	2.1	2.2	2.3	3.1	3.2	3.3	4.1	4.2
1.1 Learning/recall/executive function	1	.29***	.24**	-.25***	-.25***	-.17*	-.08	-.04	.07	.25**	.23**
1.2 Psychomotor ability/ expressive language	.28***	1	.62***	-.13	-.17*	.17*	-.19*	-.03	.02	.22**	.11
1.3 Orientation/awareness	.21**	.77	1	.05	-.01	.03	-.12	-.08	.03	.11	.02
2. Psychological Distress (alpha = .90(.90))											
2.1 Depression	-.25***	.14	-.12	1	.76***	.78***	-.16*	-.23**	-.31***	-.37***	-.37***
2.2 Anxiety	-.15*	-.11	-.11	.78***	1	.76***	-.05	-.12	-.16*	-.30***	-.28***
2.3 Stress	-.08	.10	-.12	.73***	.73***	1	-.14	-.18*	-.20*	-.40***	-.34***
3. Social Well-being (alpha = .60(.60))											
3.1 Social acceptance and integration	-.06	-.01	.11	-.17*	-.17*	-.25***	1	.52***	.25**	.12	.20
3.2 Social coherence and contribution	.13	.13	.14	-.22**	-.18*	-.17*	.54***	1	.25**	.30***	.29***
3.3 Social actualization	.00	.09	.09	-.11	-.13	-.20**	.21*	.21*	1	.08	.16
4. Mental Well-Being (alpha = .78(.71))											
4.1 Psychological well-being	.27***	.25***	.28***	-.23**	-.21**	-.27***	.08	.21*	.07	1	.56***
4.2 Positive functioning	.18*	.18*	.17*	-.32***	-.29***	-.29***	.09	.23**	.04	.63***	1

*Note*. The correlation coefficient for aging in place group are below the diagonal and for aging migrant group are above the diagonal.

*p < .05, **p < .01, ** p < .01 two-tailed.

To examine whether the predictability of cognitive function, psychological distress, and social well-being for mental well-being are similar between two types of living after the measurement invariance was verified, we combined the fit indices of the restricted and unrestricted models, as shown in Table 4.

**Table 4:** Fit indices of all models.

	Multiple-group model		Single-group model	
	Unrestricted	Restricted	Older Adult Aging in Place Group	Older Adult Migrant Group
No. of parameters	56	46	28	28
χ2	118.24	171.97	61.76	56.48
df for χ2	76	86	38	38
p-value for χ2	0.0014	< .0001	0.0087	0.0272
Scaled-χ2	118.87	166.85	65.21	54.1900
p-value for Scaled-χ2	.0012	< .0001	0.0039	0.429
SRMR	0.0653	0.1343	0.0553	0.0762
RMSEA	0.0584	0.0754	0.0620	0.0542
RMSEA, Lower limit 90%CI	0.0370	0.0581	0.0351	0.0102
RMSEA, Upper limit 90%CI	0.0780	0.0924	0.0871	0.0850
CFI	0.941	0.9185	0.9496	0.9377
TLI	0.9157	0.8774	0.9234	0.9053

The commonly reported fit indices in Table 4 indicated a good fit (i.e., the SRMR and RMSEA were less than 0.08, and the CFI and TLI were greater than or equal to 0.95). Subramaniam [32, 33] in the unrestricted model and some indices did not fit in the restricted model. Then, we tested each model separately. Both the structural model of aging in place and the aging migrant group fit the empirical data (R^2^ for aging in place group = .24, for migrant group = .42).

**Fig 2:**
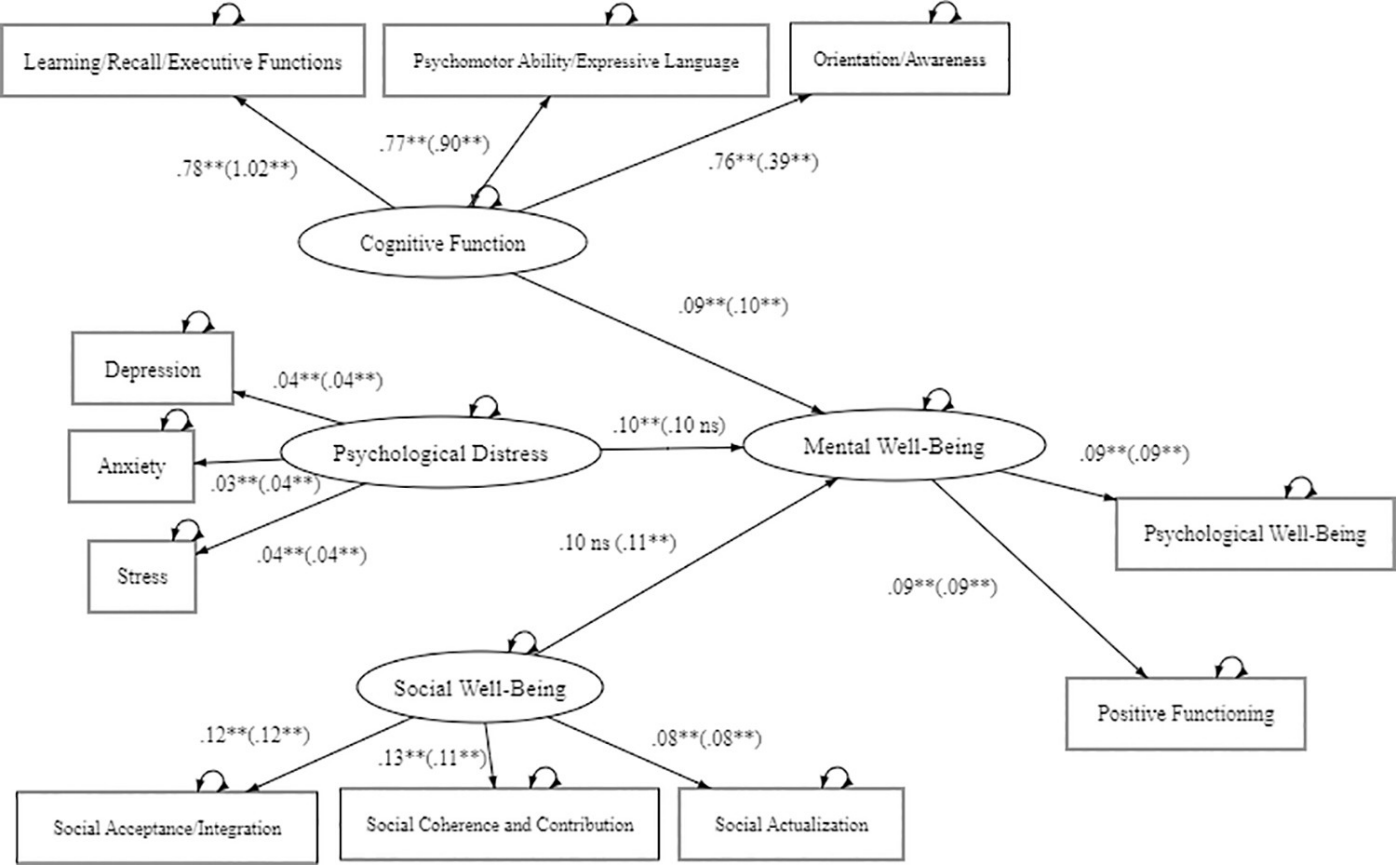
Standardized path estimates between variables for the aging in place group (n = 187) and aging migrant group (n = 147). Note the correlation coefficient for aging in place group are outside and for aging migrant group are inside the (). *p < .05, **p < .01, ** p < .01 two-tailed.

Table 5 provides the unstandardized estimates from the final restricted multi-group model. Findings revealed that for the aging in place group, psychological distress (SE = 0.08, p < .001) and cognitive function (SE = 0.07, p < .001) were significant predictors of mental well-being, but not social well-being (SE = 0.09, ns). With regard to the migrant group, psychological distress (SE = 0.12, p < .01) and social well-being (SE = 0.11, p < .001) were significant predictors of mental well-being, but not of cognitive function (SE = 0.12, ns). Fig 2 shows a standardized path estimate of the two groups.

**Table 5:** Unstandardized estimates from the single-group models.

	Aging in Place Group (n = 187)				Aging Migrant Group (n = 147)			
*Measurement models*	*λ*	SE	*t*	*p-value*	*λ*	SE	*t*	*p-value*
Cognitive function = > Learning/Recall/Executive function	3.04	0.52	5.88	< .001	3.84	1.15	3.35	< .001
Cognitive function = > Psychomotor ability/Expressive language	8.52	0.77	11.13	< .001	7.28	1.62	4.49	< .001
Cognitive function = > Orientation/Awareness	7.82	0.95	8.26	< .001	2.63	0.64	4.10	< .001
Psychological distress = > Depression	0.53	0.05	9.82	< .001	0.58	0.06	9.94	< .001
Psychological distress = > Anxiety	0.49	0.05	10.40	< .001	0.50	0.06	8.24	< .001
Psychological distress = > Stress	0.52	0.05	9.49	< .001	0.56	0.05	12.11	< .001
Social well-being = > Social acceptance and integration	0.85	0.12	7.29	< .001	0.81	0.15	5.49	< .001
Social well-being = > Social coherence and contribution	1.01	0.13	7.89	< .001	0.82	0.14	5.86	< .001
Social well-being = > Social actualization	0.26	0.07	3.41	< .001	0.26	0.08	3.21	0.001
Mental well-being = > Psychological well-being	1.00			< .001	1.00			< .001
Mental well-being = > Positive functioning = > Mental well-being	1.03	0.18	5.86	< .001	0.93	0.14	6.60	< .001
*Path model*	*B*	SE	*t*	*p-value*	*B*	SE	*t*	*p-value*
Cognitive function = > Mental well-being	0.24	0.07	3.39	< .001	0.22	0.12	1.83	ns
Psychological distress = > Mental well-being	-0.30	0.08	-3.74	< .001	-0.37	0.12	-3.07	0.002
Social well-being = > Mental well-being	0.17	0.09	1.83	ns	0.34	0.11	3.22	0.001

## Discussion

This empirical study finds that positive aging and place attachment have a significant impact on mental well-being of older adults who have resided in the same community for a long time and those who migrated to that community. These results add to the literature on aging in place by showing that the predictive model of mental well-being, which included social well-being, cognitive function, and psychological distress for both Thai older adults aging in place and migrant groups fit the empirical data. In addition, psychological distress is a crucial predictor for the mental well-being of older adults in both groups. Cognitive function is a significant predictor of mental well-being only for members of the aging in place group. Social well-being is a significant predictor of mental well-being but not of cognitive function for the aging migrant group.

The research provided evidence of a beneficial effect of reducing psychological distress for older adults in both groups. There were some similarities with the findings of Tuicomepee and colleagues [16]. Although social well-being was a significant predictor of mental well-being among Thai older adults living in the Bangkok metropolitan area, there are still some findings worth noting concerning psychological distress that affected older adults’ mental well-being. In the previous study, only depression, as a component of psychological distress, was a significant predictor of older adults’ mental well-being. For this study, all components of psychological distress significantly predicted mental well-being of older adults in both groups. One possible explanation for the different result would be the negative impact of the COVID-19 pandemic. Since Tuicomepee et al. [16] collected data before the COVID-19 pandemic, we conducted our data collection from March 2021 to February 2022. Research on the impact of the COVID-19 pandemic on older adults reported that t had significantly increased their experiences symptoms of depression, anxiety, stress) in August 2020 compared with May 2019 [36]. In Thailand, Tangthong and Manomaipiboon reported that older Thai adults had a higher prevalence (20.5%) of depression than before the pandemic, accounting for the prevalence rate of 9.6% reported in an earlier outpatient study of a similar population in the province of Songklanagarind [37]. This study suggests that local and national policies and service providers to focus on reducing symptoms caused by psychological distress for both groups, which would contribute to greater mental well-being.

Second, finding from this study highlights the role of social well-being in promoting older adults’ mental well-being in the aging migrant group, but not for the aging-in-place group. This evidence supports the integration of positive aging and Rowles’s concept of place attachment in the context of aging in place research. According to Keyes [19], social well-being is the appraisal of people’s situation and functioning in society. Rowles’s concept of place attachment underscores how the physical and social environment influence mental well-being among aging migrants. In their study the older adults who remained in their long-term homes reported higher levels of life satisfaction and emotional well-being compared to those who moved [4–5, 13]. The result points to vulnerability among older adults with migration background who experience aspects of social well-being that affect their mental well-being. Even though, the time since they migrated is more than 10 years, mean duration of years living in the current community as of 41.21± 17.56 years, the result still supports migrant studies in that social well-being is associated with migrants’ mental well-being.

There are at least two explanations for this result. One explanation is the possible heterogenous migration background of older adult migrants in this study. For instance, migrants had lived in their community from two to 70 years. Even though most people form strong community and social ties in their destination, the aging migrants in this study might not have done so. To account for the diversity in their migration background, research on social well-being among these older migrants should be analysed. Another explanation is that the COVID-19 pandemic may have caused more damage to the social networks and social support among older adult migrants than among those who were aging in place. The finding is in line with the Swiss study of Dones and Ciobanu. They found that older adult migrants residing in Switzerland reported more loneliness and worse well-being as a consequence of COVID-19 restrictions than Swiss natives did [38].

Finally, consistent with prior research [15, 21], this study found that older adults with greater cognitive function are likely to report a high level of mental well-being. However, the positive relationship between cognitive function and mental well-being was confirmed only for the aging-in-place group. This result can be explained by the role of the neighbourhood social environment. According to Finlay and colleagues, a neighbourhood that is safe and accessible can stimulate cognitive function for people who are aging in place [21], and that safety and accessibility on the neighbourhood contributes to their well-being. Neighbourhoods with strong support systems (e.g., friendly neighbours, community volunteers), which provide more opportunities for social engagement, social ties, and physical activity, can be beneficial for the cognitive function and mental well-being of people who are aging in place. For the migrant group, this study found no association between cognitive function and mental well-being in older adult migrants. While there is evidence that older migrants demonstrated poorer cognitive function than non-migrants [23], some studies indicated no association between migration and cognitive function [24]. The relationship between cognitive function and mental well-being in older adult migrants might depend on their socioeconomic condition, cultural adaptation, migrant status, and individual experiences. The result of this study indicates no association between cognitive function and mental well-being among aging migrants may be less straightforward, a finding that merits further investigation.

These findings have implications for policy makers and service providers as they consider the diverse needs of older adults. In Thailand, village health volunteers work under the supervision of the Ministry of Public Health. Each village health volunteer is selected by their community based on their public mindedness and commitment to the health of their communities. A volunteer is assigned ten households [39]. Local policies and community interventions may focus on having these volunteers facilitate social connections by strengthening their place attachment and sense of belonging among older migrants.

This study was conducted during the COVID-19 pandemic where the association between psychological distress and mental well-being for both groups might have been heightened. Another point is possible issues of bias and generalizability. This study used Quota sampling method with modest sample sizes. To mitigate some limitations related to bias and generalizability, the researchers regularly monitor the quota to ensure representative samples are being filled accurately and adjust recruitment strategies as needed.

## Conclusions

Thailand, like many countries, is experiencing a rapidly growing and increasingly diverse aging population. This study, based on concepts of positive aging and place attachment in the literature on aging in place, recommends targeted policies and practical interventions to facilitate older adults’ mental well-being. For both groups, alleviating symptoms of psychological distress for older adults can significantly improve mental well-being. For the migrant group, a significant linkage between social well-being and mental well-being was found. Accordingly, policies and community interventions for aging migrants should support their personal connection to existing networks and offer social resources to facilitate their integration into the community. For people who are aging in place, there is a significant association between cognitive function and mental well-being. This suggests that policies and community interventions should aim at maintaining their cognitive function such as lifelong learning, increased access to and safety of the neighbourhood social environment, and home modifications to create a supportive living environment.

## Supporting information

S1 Data Raw data set is available on the OSF website at https://osf.io/3ywqk/?view_only=1e14d2e1599f4cf7a408c3e04ecc6195. (TXT)
